# Simultaneous laparoscopic nephrectomy in ADPKD

**DOI:** 10.4103/0972-9941.68577

**Published:** 2010

**Authors:** Ramen Kumar Baishya, Prabhat Ranjan, Ravindra B Sabnis, Mahesh R Desai

**Affiliations:** Department of Urology, Muljibhai Patel Urological Hospital, Nadiad, Gujarat - 387 001, India

Dear Sir,

I read the interesting article ‘Concomitant laparoscopic urological procedures: Does it contribute to morbidity?’ written by Mourya *et al*.[1] Author has emphasized that simultaneous laparoscopic procedures can be done for urological diseases in selected patients with the advantages of single anaesthesia and hospital admission without increasing the morbidity. We strongly believe this point. We would like to emphasize that bilateral pre-transplant nephrectomies in autosomal dominant polycystic kidney disease is probably difficult due to large size kidney and associated infection, inflammation and adhesion. We have seen intestinal adhesions during laparoscopy for subsequent staged nephrectomy, leading to difficulty in dissection.[2] Bilateral simultaneous nephrectomy has an additional benefit of leaving the patient with minimum number of days with anephric status as renal transplantation can be done after a gap of 7-10 days of bilateral pre-transplant nephrectomies. Gill *et al*.,[3] Jenkins *et al*.,[4] Dunn *et al*.[5] have similar experience in this subject.
